# Using Medical Named Entity Recognition in Automatic ICD Prediction

**DOI:** 10.1155/bmri/6117755

**Published:** 2025-09-18

**Authors:** Mohamad Kawas, Bassel Alkhatib, Khaled Omar, Khaled Tofelia, Mayssoon Dashash, Dorota Formanowicz

**Affiliations:** ^1^ Web Science Program, Syrian Virtual University, Damascus, Syria, svuonline.org; ^2^ Artificial Intelligence Department, Information Technology Engineering College, Damascus University, Damascus, Syria, damascusuniversity.edu.sy; ^3^ Pediatric Dentistry Department, Dentistry College, Damascus University, Damascus, Syria, damascusuniversity.edu.sy

**Keywords:** automatic ICD coding, BERT Medical NER, ClinicalBERT

## Abstract

The International Classification of Diseases (ICD) serves as a standard in medical coding. Researchers in artificial intelligence, including those focused on natural language processing and machine learning, have made a significant effort to build and develop automatic ICD encoding systems and algorithms. Many algorithms have been developed to implement automatic ICD encoding, but almost all of these algorithms depended on the raw text input without taking into consideration the important medical entities in this input. In this paper, we propose an algorithm for automatically predicting ICD codes based on patient claims. Our algorithm contains several steps for finding the most relevant ICD codes. Primarily, our proposed algorithm employs medical named entity recognition (NER) to find the most important medical entities in a patient claim. For this purpose, the Medical NER model was used based on the BERT model. Next, the algorithm generates embeddings for the extracted entities using the ClinicalBERT model. To identify the most relevant ICD code, the algorithm creates embeddings for an ICD catalog, which contains various information such as chapter descriptions, long descriptions, short descriptions, and ICD codes. The embedding process is primarily based on the long descriptions, and the results are stored in a local database that contains embedding vectors and corresponding mapped ICD codes. The final step of the algorithm calculates the cosine similarity between the embedding vector generated from the patient complaint and the ICD long description vectors. The strength of this new algorithm is that it first detects the medical entities in the textual input and then predicts the most similar ICD codes. Also, our developed algorithm does not need such huge data for training. We tested the developed algorithm on a medical dataset, and the results indicate that the proposed method is highly efficient, achieving a precision rate of approximately 90%.

## 1. Introduction

The integration of computer‐supported technology and medicine has tremendous potential for creating positive outcomes. Medical health is expected to enhance healthcare by providing access to high‐quality medical information, enabling patients to take better care of their health and avoid neglecting medical issues. This information can be easily accessible on mobile phones or devices connected to the internet, anytime and anywhere.

Also, AI technology is developing at a rapid rate, including machine learning technology, which has now become very popular and is utilized in all fields. Additionally, word/sentence embedding models, which have now become popular and free, can be utilized in a huge range of applications.

Many medical encoding models have been built and used in the last years, and the main important thing is that these models have been trained on a huge dataset, which in turn affects the accuracy and the bias of these models.

The International Classification of Diseases (ICD) serves a broad range of uses globally and provides critical knowledge on the extent, causes, and consequences of human disease and death worldwide via data that is reported and coded with the ICD. Clinical terms coded with ICD are the main basis for health recording and statistics on disease in primary, secondary, and tertiary care, as well as on cause of death certificates. These data and statistics support payment systems, service planning, administration of quality and safety, and health services research. Diagnostic guidance linked to categories of ICD also standardizes data collection and enables large‐scale research.

Automatic disease ICD prediction has become a crucial and significant issue in the medical industry and medical insurance. ICD [1, 2] is a popular medical ontology [3], and it is a healthcare classification system maintained by the World Health Organization. All diseases and health statuses are classified according to certain rules and uniquely identified by character codes.

Usually, the ICD coding is an essential step in the healthcare process and applications. The problem of ICD coding is that there are many coding errors made by specialized persons based on guidelines for choosing the most suitable ICD codes according to patient diagnosis, and this, in turn, causes a lot of problems in detecting the specific diagnosis of the patient’s disease. In the following, we will explain the most common errors in the coding process [4]:

• Maybe doctors use abbreviations in writing diagnoses; this in turn leads to incorrect matching in ICD codes.
• In many cases, the one diagnosis may relate to more than one ICD code.
• In many cases, two or three diagnoses may relate to the same ICD code.

To avoid such errors, many researchers’ efforts were adapted to automatic ICD coding; many algorithms have been developed to implement automatic ICD coding.

The essential problems of the current developed algorithms of automatic ICD coding are that these algorithms need a huge dataset to be trained on and also the precision rate of these algorithms; thus, we proposed a new algorithm with a high accuracy rate and without any need for a dataset to be trained on by using transfer learning technology.

Named entity recognition (NER) [5] is a technology for detecting the entities in a specific domain; it has been widely used in many use cases such as text summarization, document classification, document clustering, text meaning analysis, and nowadays, the NER libraries are popular and domain‐oriented. The utilization of NER in textual data processing tasks reduces the huge volumes of text and gets rid of useless textual information because the NERs pick up the important terms and concepts of the domain. NERs are constructed based on statistical methods and language‐based rules.

Text embedding [6] is the process of converting text from textual data format to *n*‐dimensional numeric vector format that the importance of embedding comes from the fact that all of machine learning models that used for prediction and classification and regression take as input numeric data format to understand the textual input; the embedding is done by using pretrained models in the domain; many models have been developed to be used in embedding; the embedding models are domain oriented; this means that we cannot use a generic model for medical data embedding; in the medical domain, a huge number of models have been developed to perform medical text embedding; the following table describes the most popular medical embedding models:

• PubMedBERT‐base: model fined‐tuned using sentence transformers. It maps sentences and paragraphs to a 768‐dimensional dense vector space and can be used for tasks like clustering or semantic search [7].
• Clinical‐BR‐Mistral‐7B‐v0.2 is a fine‐tuned language model specifically designed for generating clinical notes in Portuguese [8].
• MedEmbed: MedEmbed is a family of embedding models fine‐tuned specifically for medical and clinical data [9].

The following of this paper will contain the following sections: the related work in ICD automatic prediction, proposed algorithm for ICD prediction, evaluation, and conclusion.

## 2. Related Work

Many algorithms have been developed by researchers to predict the ICD codes based on the textual data; in general, this textual data is related to the patient’s records. Almost all ICD prediction algorithms include textual feature extraction steps before building the algorithm. We will mention the general techniques that are applied to the textual data; we can classify these techniques as follows:

• Stop words removing: in this step, the stop words are removed from the textual input; these words are useless and removing them will reduce the complexity of the text manipulation; the stop words list varies from language to language; it is a language‐oriented feature.
• Text stemming: in this step, all the words in the textual input are returned to their roots using the popular natural language processing (NLP) stemmers; the implementation of the stemming algorithms is varied according to the language rules. Stanford stemmer [10] is one of the most popular stemmers for the English language.
• Term frequency–inverse document frequency (TF/IDF) evaluates the importance of the word in a document or corpus [11]; the TF is a measure of the importance of the term in one document, and IDF is a measure of the importance of the term in all corpus documents.
• Bag‐of‐words (BoW): a vocabulary of unique words is created, where each word represents an independent and discriminative feature [12]. BoW can also be used effectively in document retrieval tasks.
• *N*‐gram is the contiguous sequence of words, where *N* may be “1,” “2,” “3,” and so on. One word sequence is called 1‐gram (or unigram); the sequence of two words is called 2‐gram (or bigram), the sequence of three words is called 3‐gram (trigram), and so on [12].
• Word2Vec is a method to construct embeddings using two models: skip‐gram and continuous bag of words (CBOW). The skip‐gram model learns from the existing words available in a sentence to predict the next word, whereas the CBOW model uses the neighboring word to predict the next word [13].
• Doc2Vec and Paragraph2vec are variants of word2vec. They focus on predicting words in the document or paragraph. Doc2vec creates a numeric representation of a document irrespective of its length [14].
• Global vectors (GloVe) is another commonly used word embedding method that derives the relationship between the words from the global corpus statistics [15].
• FastText builds on a specific limitation of word2vec and GloVe. It can handle out‐of‐vocabulary terms by extending the skip‐gram model with internal subword information [16].

Also, ICD coding algorithms can be classified according to the methodology into the following categories:

• Rule‐based ICD coding algorithms: These systems rely on a predefined set of rules, keywords, and phrases mapped to specific ICD codes. They often use terminologies like SNOMED CT or MeSH and may involve simple if‐then logic or Boolean operators [17]; these types of algorithms have many limitations such as the following:
  o. Brittleness: cannot handle synonyms, abbreviations, or variations in language.
  o. Low recall: misses complex cases where the diagnosis is implied rather than stated explicitly.
  o. High maintenance: requires constant manual updates by experts to remain current.
• Traditional machine learning ICD coding algorithms: as researcher in [18] employed text classification to categorize clinical narratives into various categories using machine learning approaches including supervised, unsupervised, semisupervised, the most popular ones based on supervised learning algorithms as researcher in [19] the clinical narratives collected from hospitals are labeled by domain experts into specific categories.
• Deep learning ICD coding algorithms: these algorithms are a type of machine learning technique that utilizes a multilayered neural network architecture to learn the hierarchical representation of data. Deep learning models have demonstrated successful results in many NLP tasks such as language translation [20], image captioning [21], and sentiment analysis [22] The performance of machine learning methods heavily depends on data representation (or features) on which they are applied. Therefore, much of the effort deploying machine learning algorithms goes into the design of a preprocessing pipeline and data transformation.
• Hybrid ICD coding algorithms: these algorithms use a mixed methodology from various algorithm types that there are many algorithms that use rule‐based ICD coding with deep learning ICD codes and also many algorithms that use text classification with rules for detecting the right ICD codes according to the input.

Many systems and algorithms have been developed to generate or predict the ICD codes automatically based on medical texts, as the accuracy and efficiency of manual ICD coding have always been a concern of clinical practice. In [23], researchers have summarized the complete workflow of assigning ICD codes manually, which is a long and hard procedure and contains many errors. To get rid of human errors in ICD coding, the efforts of researchers have proposed some automatic or semiautomatic ICD classification systems, but the datasets have generally been small and domain‐specific. In [24], researchers developed a knowledge attention‐based deep learning framework called KAICD for automatic ICD coding. KAICD makes full use of the clinic notes and the ICD titles. In [25], researchers developed an automated ICD coding system via machine learning that focuses on heart diseases, consisting of three models. The first one is a BERT variant module responsible for encoding clinical text. The second is a word2vec module for encoding code titles, and the third is a label‐attention module for integrating the embedding of clinical text and code titles. In [26], researchers developed the task of automatically classifying medical documents into the taxonomy of the ICD by using deep neural networks. In [27], researchers propose a hierarchical deep learning model with an attention mechanism that can automatically assign ICD diagnostic codes given written diagnoses. They utilize character‐aware neural language models to generate hidden representations of written diagnosis descriptions and ICD codes and design an attention mechanism to address the mismatch between the number of descriptions and corresponding codes.

In [28], researchers developed an automatic ICD prediction algorithm based on textual medical data from the outpatient department (OPD) of a university hospital; a NLP technique was used (word embedding, Word2Vector) to process the data, and then, a deep learning–based convolutional neural network (CNN) model was created based on the OPD patient information.

Also, many researchers try to identify the patient disease ICD based on identifying the medical concepts in the patient records; for this type of algorithms, concept‐based features are extracted using either medical ontology such as SNOMED‐CT2 [29].

In general, artificial intelligence, including machine learning, has been utilized in the medical sector to predict disease. In [30], researchers tried to use CNN to develop models for disease prediction, and in [31], researchers proposed a hybrid mathematical model that combines features from supervised learning and some other features from unsupervised learning to obtain an easy and robust algorithm in terms of prediction and computation for breast cancer prediction. In [32], researchers used the Medical Information Mart for Intensive Care (MIMIC III) dataset and applied four different machine learning algorithms: decision tree classifier, random forest, artificial neural network (ANN), and logistic regression for predicting the length of stay of patients (construction workers) during the COVID pandemic. In [33], a new system for automatic Alzheimer’s disease detection was proposed by utilizing graph Fourier transform from EEG signals. In [34], knowledge graph enrichment from clinical narratives using NLP, NER, and biomedical ontologies for healthcare applications was discussed.

In [35], researchers used NLP steps and word embedding to predict the ICD codes based on textual input.

In, researchers made a systematic literature review of automated ICD coding and classification systems using discharge summaries.

In Table 1, we will compare our developed algorithm with the categories of the current algorithms.

**Table 1:** Comparison of the developed algorithm with existing algorithms.

**Algorithm category**	**Manual feeding**	**Training data**
Rule‐based ICD coding algorithms	Need continuous feeding to add new rules to detect the ICD codes	Need a huge data which must extract from domain experts and then need to format this data in rules
Traditional machine learning ICD coding algorithms	Need continuous data updating to retrain the models to extract ICD codes	Need a huge labeled data to train the models to extract ICD codes
Deep learning ICD coding algorithms	Needs continuous data representation to extract features that will be used in model training	Need huge features which must be extracted from existing datasets
Our developed algorithm	No need from manual feeding	No need for data for training

## 3. Materials and Methods

**Figure 1:**
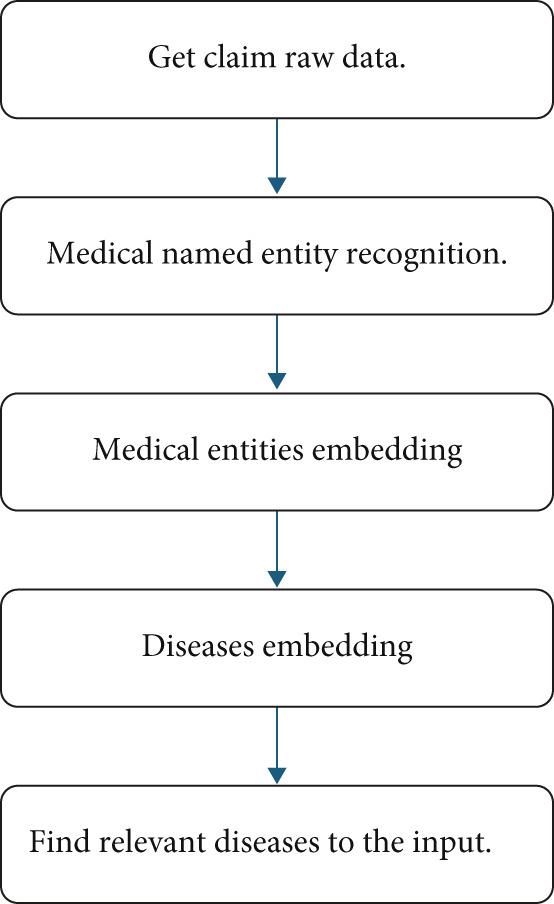
Proposed algorithm steps.

Figure 1 contains the main steps of our proposed algorithm.

We will talk in detail about each step in the following.

### 3.1. Get Claim Raw Data

In this step, the input is loaded to the system; the input is textual raw data which contains medical information about the patient status; this information contains symptoms, body parts, diseases, and other information.

### 3.2. Medical NER

The goal of this step is to detect the most important information in the claim textual data that NER is essentially a technology that enables automatic identification and classification of entities—such as patient names, medical conditions, symptoms, diseases, medications, and procedures—in a sea of unstructured text data. In order to detect these entities, we have used a Medical NER model which is fine‐tuned on the BERT model to recognize 41 medical entities [36]. This model is a fine‐tuned version of DeBERTa on the PubMed dataset [37].

We are focusing our algorithm on the following entity types:

• Biological structures
• Symptoms
• Age
• Gender
• Diseases

**Figure 2:**
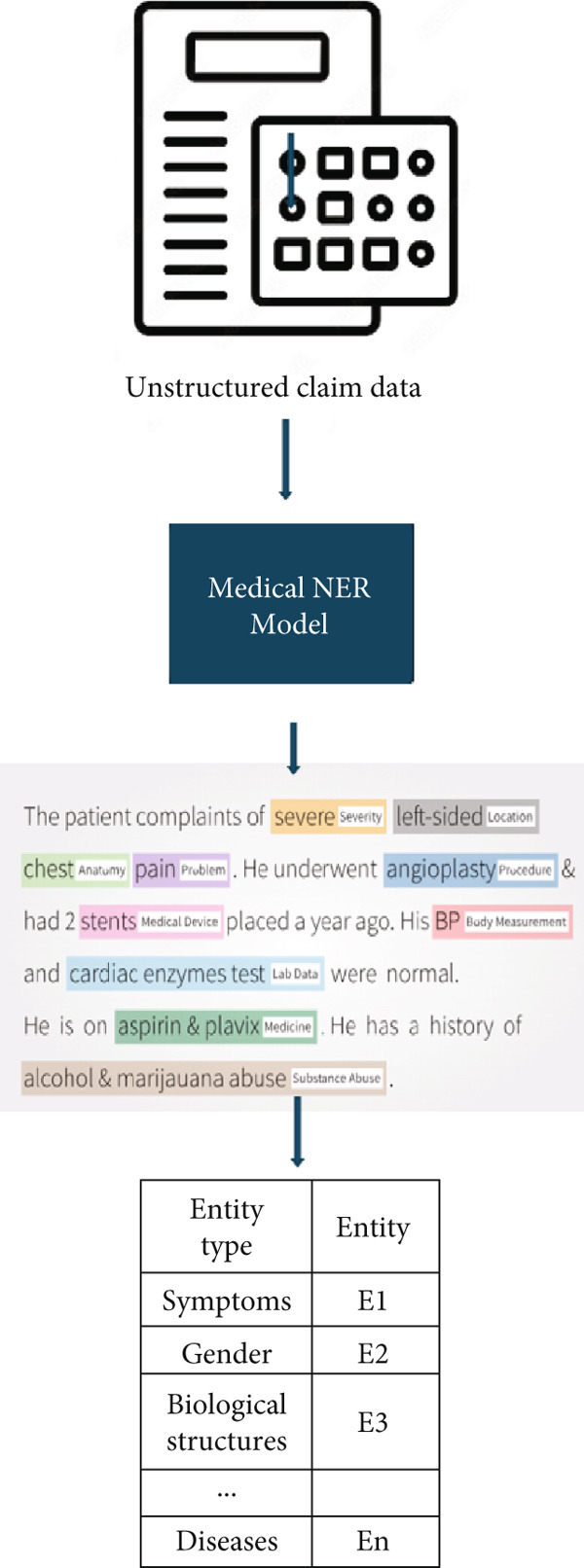
Medical named entity recognition process.

We have used this model as it trained and fine‐tuned on BERT model which contains 110M parameters. By the end of this step, the claim unstructured text data is converted to a list of entities. The following figure describes the NER process; Figure 2 contains the steps of medical NER from claim raw data.

As we see from the figure above, the input of this step is unstructured textual data and the output of the step is a list of medical entities.

### 3.3. Medical Entity Embedding

In this step, the extracted entities from the previous step are converted to vector embeddings. The embedding is done by using the ClinicalBERT [38] model. ClinicalBERT learns deep representations of clinical text. These deep representations can be used to uncover clinical insights, such as predictions of disease, relationships between treatments and outcomes, or summaries of a large volume of texts. It was trained on a large multicenter dataset with a large corpus of 1.2B words of diverse diseases. This model training used a batch size of 32, a maximum sequence length of 256, and a learning rate of 5*e* − 5 for pretraining our models.

By the end of this step, the detected entities will be converted to vector representations.

**Figure 3:**
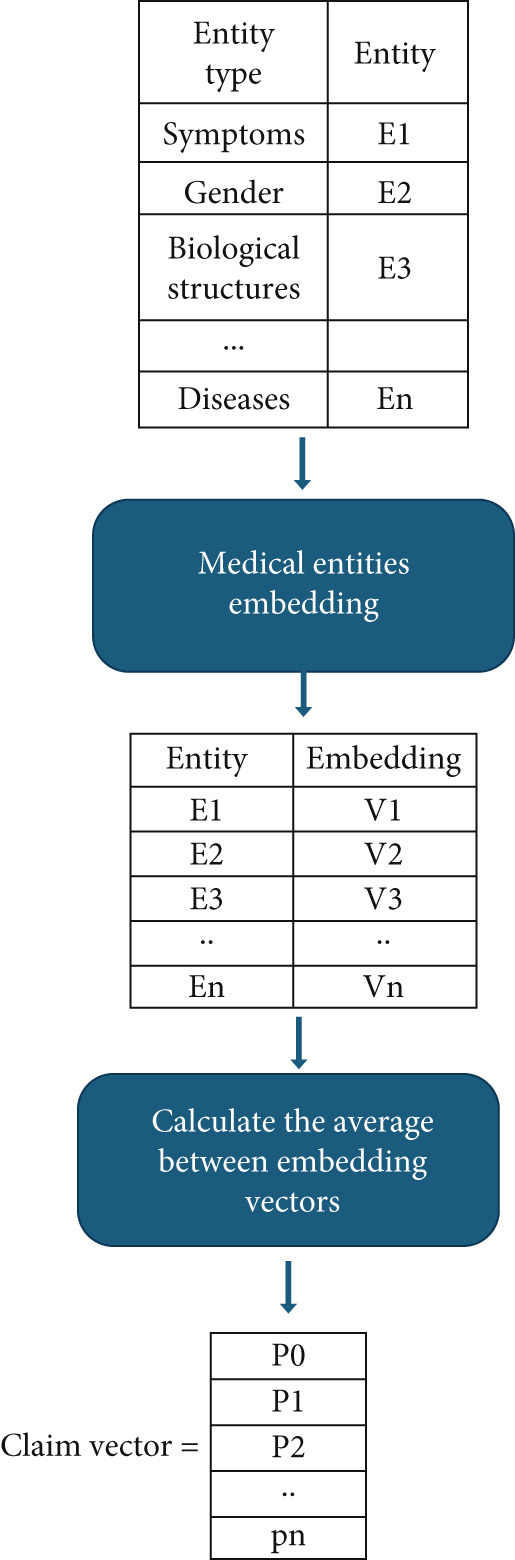
Medical entity embedding.

For example: detected medical entities: [cough, runny nose, headache, sneezing…] will be converted to [embed‐vec1, embed‐vec2, embed‐vec3., embed‐vec4…]. Then, after finishing the conversion of entities to vectors, we will take the average of all these vectors representing the relative information from all of them. Figure 3 contains the steps of the medical entity embeddings.

### 3.4. Disease Embedding

In this step, the diseases are embedded based on the ICD catalog (the long description of the ICD code is embedded); that after finishing this step, all the ICD‐10 codes are converted to numeric numbers based on text description for every ICD code; that the ICD catalog [28] contains short description and long description; this encoding step depends on ICD long description and converts each ICD long description to numeric vector using ClinicalBERT model; after finishing this step, all ICD long descriptions are converted to numeric vectors and stored into local database (developed system database); Table 2 contains the information of the ICD‐10 codes with the long description embedding vectors.

**Table 2:** ICD code embedding.

**ICD-10 code**	**ICD-10 long description embedding vector**
Code1	V1
Code2	V2
..	..
CodeN	Vn

### 3.5. Find Relevant Diseases to the Input

In this step, the algorithm tries to find the most relevant ICD codes (most similar ICD codes) to the input as the following:

• By calculation, the similarity between input‐generated average vector (which was generated in Step 3)
• The ICD codes’ descriptions (which are already encoded and converted to numeric vectors and stored into system database) based on the following formula (Equation 1) which calculates the cosine similarity between vectors [39]:

cosθ=∑i=1nAiBi∑Ai2∑Bi2

where *A* is the generated average vector of the claim textual input and *B* is the numeric vector of the ICD long description.

So the similarity score will be as the following:

Similarity score input vector,ICD codes=cosine−similarity input vector,ICD codes

## 4. Results and Discussion

### 4.1. Dataset

As we mentioned in the proposed algorithm, the second layer calculates the similarity between the input (patient complaint) and the local database records, which include labeled patient complaints, which means that each record contains a patient complaint and its validated ICD code. This validation is done by the medical encoders. The local database contains 10,000 records of labeled and valid data.

### 4.2. Evaluation Metric

The data is divided into two parts, each part contains 5000 records; the first set is used in training the algorithm, and the second set is used in validation. We have used the precision metric for evaluating our development to calculate the accuracy of the developed algorithm because we are depending on our evaluation of human ICD labeled data and measuring our developed algorithm’s accuracy by comparing results returned by our algorithm with the actual ICD codes. The precision formula is defined as follows:

Precision=extracted ICD codes∩ICD codesextracted ICD codescosΘ=A∗BA∗B

where extracted ICD codes are codes that are returned by the developed algorithm and ICD codes are the actual codes which are related to the patient complaint.

After testing the algorithm on the testing dataset (5000) records, the precision is as shown in Table 3:

**Table 3:** Precision result.

**Precision**
90.03

As a comparison of the current developed algorithm with the existing algorithm for ICD prediction, our developed algorithm’s performance is greater than these algorithms because of its two layers. In the first layer, the algorithm detects the medical entities using the Bert‐Medical NER model (which is fine‐tuned version of DeBERTa on the PubMed dataset) and excludes the useless textual data, which will highly affect the accuracy of our algorithm. Then, these entities are used for ICD prediction by using ClinicalBERT (which was trained on a large multicenter dataset with a large corpus of 1.2B words of diverse diseases); for performance, our proposed model was in real time because we have used a vector database to get the relevant ICDs for the input, instead of memory allocation of the similarity.

## 5. Conclusion

In this study, we presented a multilayer algorithm for ICD prediction that utilizes word embedding and NLP. Our algorithm employs PubMedBERT, a pretrained model built from abstracts in PubMed and full‐text articles from PubMedCentral, to achieve medical text word embeddings. This model demonstrates state‐of‐the‐art performance across various biomedical NLP tasks and currently holds the top score on the biomedical language understanding and reasoning benchmark. Our developed algorithm layers significantly enhance accuracy by predicting the ICD code related to patient complaints through two primary similarity layers. The first layer assesses similarity between patient complaint and ICD long description, while the second layer evaluates the similarity between the patient complaint and local database records. We established a certainty factor of 0.3 for the first layer and 0.7 for the second layer to generate the final similarity results. We tested our developed algorithm on a dataset comprising 1000 records, which was split into two sets: 5000 records for training and 5000 records for testing. The developed algorithm successfully generated ICD codes relevant to patient complaints with an impressive accuracy rate of approximately 90%. The current version of this algorithm is designed to be utilized by any system, as we have developed it as a standalone service API that can be called from other systems. In the future, we aim to further enhance the accuracy of our algorithm by implementing hierarchical classification in machine learning and employing modeling concepts using medical ontologies to discover relationships among various diseases.

This developed algorithm has one limitation: that this algorithm’s accuracy rate cannot be 100% because it is an automatic ICD coding algorithm decision support, and the final decision will be taken by the clinical expert.

## Disclosure

All coauthors have seen and agree with the contents of the manuscript. We certify that the submission is original work and is not under review at any other publication.

## Conflicts of Interest

The authors declare no conflicts of interest.

## Funding

No funding was received for this manuscript.

## Data Availability

Data sharing is not applicable to this article as no datasets were generated or analyzed during the current study.

## References

[1] Free 2024 ICD-10-CM Codes, 2024, https://www.icd10data.com/ICD10CM/Codes.

[2] Harrison J. E. , Weber S. , Jakob R. , and Chute C. G. , ICD-11: An International Classification of Diseases for the Twenty-First Century, BMC Medical Informatics and Decision Making. (2021) 21, no. Supplement 6, 10.1186/s12911-021-01534-6, 34753471. PMC8577172 34753471

[3] Guarino N. , Oberle D. , and Staab S. , What Is an Ontology?, Handbook on Ontologies, 2009, Springer, 1–17, 10.1007/978-3-540-92673-3_0.

[4] Burks K. , Shields J. , Evans J. , Plumley J. , Gerlach J. , and Flesher S. , A Systematic Review of Outpatient Billing Practices, SAGE Open Medicine. (2022) 10, 20503121221099021, 10.1177/20503121221099021, 35646364. 35646364 PMC9134459

[5] Named Entity Recognition (NER) , ENCORD, https://encord.com/glossary/ner-definition/.

[6] Mansurova M. , Text Embeddings: Comprehensive Guide - TDS Archive - Medium. Medium, 2024, https://medium.com/data-science/text-embeddings-comprehensive-guide-afd97fce8fb5.

[7] NeuML/pubmedbert-base-embeddings · Hugging Faces (2001, January 26), https://huggingface.co/NeuML/pubmedbert-base-embeddings.

[8] pucpr-br/Clinical-BR-Mistral-7B-v0.2 · Hugging Face, https://huggingface.co/pucpr-br/Clinical-BR-Mistral-7B-v0.2.

[9] abhinand/MedEmbed-base-v0.1 · Hugging Face, (2024, October 21). https://huggingface.co/abhinand/MedEmbed-base-v0.1.

[10] Stemming and Lemmatization, https://nlp.stanford.edu/IR-book/html/htmledition/stemming-and-lemmatization-1.html.

[11] TF-IDF — Term Frequency-Inverse Document Frequency - LearnDataSCI, https://www.learndatasci.com/glossary/tf-idf-term-frequency-inverse-document-frequency/.

[12] Brownlee J. , A Gentle Introduction to the Bag-of-Words Model. MachineLearningMastery.com, 2019, https://machinelearningmastery.com/gentle-introduction-bag-wordsmodel/.

[13] Gensim: Topic Modelling for Humans, https://radimrehurek.com/gensim/models/doc2vec.html.

[14] Shperber G. , A Gentle Introduction to Doc2Vec - Wisio - Medium, 2019, Medium. https://medium.com/wisio/a-gentle-introduction-to-doc2vec-db3e8c0cce5e.

[15] Pennington J. , Socher R. , and Manning C. , Glove: Global Vectors for Word Representation, Proceedings of the 2014 conference on empirical methods in natural language processing (EMNLP), 2014, Association for Computational Linguistics, 10.3115/v1/D14-1162.

[16] Mojumder P. , Hasan M. , Hossain M. F. , and Hasan K. A. , A Study of Fasttext Word Embedding Effects In Document Classification in Bangla Language, Proceedings of the International Conference on Cyber Security and Computer Science, 2020, Springer International Publishing, 441–453, 10.1007/978-3-030-52856-0_35.

[17] Stanfill M. H. , Williams M. , Fenton S. H. , Jenders R. A. , and Hersh W. R. , A Systematic Literature Review of Automated Clinical Coding and Classification Systems, Journal of the American Medical Informatics Association. (2010) 17, no. 6, 646–651, 10.1136/jamia.2009.001024, 2-s2.0-78650497348, 20962126. 20962126 PMC3000748

[18] Kaur R. and Ginige J. A. , Comparative Analysis of Algorithmic Approaches for Auto-Coding With ICD-10-AM and ACHI, Studies in Health Technology and Informatics. (2018) 252, 73–79, 30040686. 30040686

[19] Mujtaba G. , Shuib L. , Idris N. , Hoo W. L. , Raj R. G. , Khowaja K. , Shaikh K. , and Nweke H. F. , Clinical Text Classification Research Trends: Systematic Literature Review and Open Issues, Expert Systems with Applications. (2019) 116, 494–520, 10.1016/j.eswa.2018.09.034, 2-s2.0-85053829021.

[20] Zhang J. and Zong C. , Deep Neural Networks in Machine Translation: An Overview, IEEE Intelligent Systems. (2015) 30, no. 5, 16–25, 10.1109/MIS.2015.69, 2-s2.0-84941213571.

[21] LeCun Y. , Bengio Y. , and Hinton G. , Deep Learning, Nature. (2015) 521, 10.1038/nature14539, 2-s2.0-84930630277. 26017442

[22] Socher R. , Perelygin A. , Wu J. , Chuang J. , Manning C. D. , Ng A. , and Potts C. , Recursive Deep Models for Semantic Compo-sitionality Over a Sentiment Treebank, Proceedings of the 2013 Conference on Empirical Methods in Natural Language Processing, 2013, Association for Computational Linguistics, 1631–1642, http://aclweb.org/anthology/D13-1170.

[23] Kaur R. , Ginige J. A. , and Obst O. , A Systematic Literature Review of Automated ICD Coding and Classification Systems Using Discharge Summaries, 2021, https://arxiv.org/abs/2107.10652.

[24] Wu Y. , Zeng M. , Fei Z. , Yu Y. , Wu F. X. , and Li M. , KAICD: A Knowledge Attention-Based Deep Learning Framework for Automatic ICD Coding, Neurocomputing. (2022) 469, 376–383, 10.1016/j.neucom.2020.05.115.

[25] Zhao S. , Diao X. , Xia Y. , Huo Y. , Cui M. , Wang Y. , Yuan J. , and Zhao W. , Automated ICD Coding for Coronary Heart Diseases by a Deep Learning Method, Heliyon. (2023) 9, no. 3, 14037, 10.1016/j.heliyon.2023.e14037, 36938427. PMC10018467 36938427

[26] Moons E. , Khanna A. , Akkasi A. , and Moens M.-F. , A Comparison of Deep Learning Methods for ICD Coding of Clinical Records, Applied Sciences. (2020) 10, no. 15, 10.3390/app10155262.

[27] Xie P. , Shi H. , and Zhang M. , A Neural Architecture for Automated ICD Coding, Proceedings of the 56th Annual Meeting of the Association for Computational Linguistics (Volume 1: Long Papers), 2018, Association for Computational Linguistics, 10.18653/v1/P18-1098.

[28] Masud J. H. B. , Kuo C. C. , Yeh C. Y. , Yang H. C. , and Lin M. C. , Applying Deep Learning Model to Predict Diagnosis Code of Medical Records, Diagnostics. (2023) 13, no. 13, 10.3390/diagnostics13132297, 37443689. PMC10340491 37443689

[29] Koné C. , Babri M. , and Rodrigues J. , SNOMED CT: A Clinical Terminology but Also a Formal Ontology, Journal of Biosciences and Medicines. (2023) 11, no. 11, 326–333, 10.4236/jbm.2023.1111027.

[30] Mohanty B. C. , Subudhi P. K. , Dash R. , and Mohanty B. , Feature-Enhanced Deep Learning Technique With Soft Attention for MRI-Based Brain Tumor Classification, International Journal of Information Technology. (2024) 16, no. 3, 1617–1626, 10.1007/s41870-023-01701-0.

[31] Ismael S. A. A. , Mohammed A. , and Hefny H. , An Enhanced Deep Learning Approach for Brain Cancer MRI Images Classification Using Residual Networks, Artificial Intelligence in Medicine. (2020) 102, 101779, 10.1016/j.artmed.2019.101779, 31980109. 31980109

[32] Samy S. S. , Karthick S. , Ghosal M. , Singh S. , Sudarsan J. S. , and Nithiyanantham S. , Adoption of Machine Learning Algorithm for Predicting the Length of Stay of Patients (Construction Workers) During COVID Pandemic, International Journal of Information Technology. (2023) 15, no. 5, 2613–2621, 10.1007/s41870-023-01296-6, 37360312. PMC10250170 37360312

[33] Sharma R. and Meena H. K. , Graph Based Novel Features for Detection of Alzheimer’s Disease Using EEG Signals, Biomedical Signal Processing and Control. (2025) 103, 107380, 10.1016/j.bspc.2024.107380.

[34] Thukral A. , Dhiman S. , Meher R. , and Bedi P. , Knowledge Graph Enrichment From Clinical Narratives Using NLP, NER, and Biomedical Ontologies for Healthcare Applications, International Journal of Information Technology. (2023) 15, no. 1, 53–65, 10.1007/s41870-022-01145-y.

[35] Albokae N. , AlKhtib B. , and Omar K. , Hybrid Method for ICD Prediction Using Word Embedding and Natural Language Processing, Proceedings of the 2023 24th International Arab Conference on Information Technology (ACIT), 2023, IEEE, 10.1109/ACIT58888.2023.10453813.

[36] dslim/bert-base-NER · Hugging Faces, 2001, https://huggingface.co/dslim/bert-base-NER.

[37] Papers With Code - Pubmed Dataset, https://paperswithcode.com/dataset/pubmed.

[38] medicalai/ClinicalBERT Hugging Face, https://huggingface.co/medicalai/ClinicalBERT.

[39] Lahitani A. R. , Permanasari A. E. , and Setiawan N. A. , Cosine Similarity to Determine Similarity Measure: Study Case in Online Essay Assessment, Proceedings of the 2016 4th International Conference on Cyber and IT Service Management, 2016, IEEE, 10.1109/CITSM.2016.7577578, 2-s2.0-84994086881.

